# Preparation and in vitro release kinetics of ivermectin sustained-release bolus optimized by response surface methodology

**DOI:** 10.7717/peerj.5418

**Published:** 2018-07-31

**Authors:** Xiangchun Ruan, Xiuge Gao, Ying Gao, Lin Peng, Hui Ji, Dawei Guo, Shanxiang Jiang

**Affiliations:** 1 College of Veterinary Medicine, Nanjing Agricultural University, Nanjing, Jiangsu, China; 2 College of Animal Science and Technology, Anhui Agricultural University, Hefei, Anhui, China

**Keywords:** Ivermectin, Bolus, Dissolution kinetic model, Korsmeyer–Peppas, Sustained-release

## Abstract

Sustained-release formulations of ivermectin (IVM) are useful for controlling parasitic diseases in animals. In this work, an IVM bolus made from microcrystalline cellulose (MCC), starch and low-substituted hydroxypropyl cellulose (LS-HPC) was optimized by response surface methodology. The bolus was dissolved in a cup containing 900 mL of dissolution medium at 39.5 °C, under with stirring at 100 rpm. A quadratic model was formulated using analysis of variance according to the dissolution time. The optimized formulation of the bolus contained 8% MCC, 0.5% starch, and 0.25% LS-HPC. The length, width, and height of the prepared IVM bolus were 28.12 ± 0.14, 16.1 ± 0.13, and 13.03 ± 0.05 mm, respectively. The bolus weighed 11.4842 ± 0.1675 g (with a density of 1.95 g/cm^3^) and contained 458.26 ± 6.68 mg of IVM. It exhibited in vitro sustained-release for over 60 days, with a cumulative amount and percentage of released IVM of 423.72 ± 5.48 mg and 92.52 ± 1.20%, respectively. The Korsmeyer–Peppas model provided the best fit to the dissolution release kinetics, exhibiting an *R*^2^ value close to 1 and the lowest Akaike Information Criterion among different models. The parameter *n* (0.5180) of the Korsmeyer–Peppas model was between 0.45 and 0.89. It was demonstrated that the release mechanism of the IVM bolus followed a diffusive erosion style.

## Introduction

In vitro drug release from a dosage form is a valuable tool in the development of new pharmaceutical formulations. Ivermectin (IVM), an antiparasitic macrolide, is widely used in cattle, sheep, pigs, and other animals. Long-acting or sustained-release formulations of IVM have been authorized for its use in animals in several countries (Martinez, Lindquist & Modric, 2010). IVM can be incorporated into a soluble silicate glass ingredient to form a sustained-release bolus. The release profile of the bolus affects the bioavailability and clinical effects of the active ingredient. Therefore, the evaluation of the bolus release profile is an important step in the development of new IVM formulations.

Soluble glasses are biomaterials with good biocompatibility, widely used in cosmetic (Shimono et al., 1998) and biomedical (Alekseeva et al., 2012; Bitar et al., 2005) industries. Once implanted in the body, some soluble silicate glasses can bond to bone and muscles, and this behavior is referred to as bioactivity and good biocompatibility (Hench, 1998; Hench & Polak, 2002). Soluble silicate glass (one component of the bolus) is a biomaterial with good biocompatibility, and is not toxic to animals. It has a porous structure on the surface, so it can readily attach to the gastrointestinal tract to reduce the irritation by parasites. Thus, soluble silicate glass within a bolus given by oral administration appears to be a useful approach. The first bioactive glass was developed by Larry Hench at the University of Florida in 1969 (Hench, 2006). A bolus formulated with soluble glass containing trace elements was used for dietary supplementation in animals by Allen and colleagues (Allen et al., 1979). The tissue-regeneration ability of bioactive glasses has also been demonstrated (Hench, 1998; Hench & Polak, 2002). Soluble silicate glass within a bolus given by oral administration to animals was released and attached to gastrointestinal tissue. It was adapted to the physiological environment and should provide long-lasting repair. So, it could aid healing of gut damage caused by parasites. Although boluses formulated with metallic or plastic shells (Anderson et al., 1980; Jones & Bliss, 1983) can remain in the animal body after long-term use, the IVM bolus formulated with soluble glass can be readily absorbed or excreted, reducing potential risks to animals.

Response surface methodology (RSM) is a set of statistical and mathematical techniques frequently applied in nanotechnology, chemistry, and medicine (Asfaram et al., 2015; Belwal et al., 2016; Dharma et al., 2016; Maran et al., 2017; Oliveira et al., 2016) to build a model for the optimization of variable parameters in systems involving complex interactions (Homayoonfal, Khodaiyan & Mousavi, 2015). The modeling process involves some runs to optimize the individual parameters using the Box–Behnken design. Generally, analysis of variance (ANOVA) is performed for testing the statistical accuracy of a quadratic polynomial fitted to the experimental data. The quadratic regression model provides the coefficient of determination (*R*^2^) for the response values, which can then be attributed to the identified independent variables. A non-significant lack-of-fit for all variables indicates that the polynomial model provides a statistically accurate representation of the responses. In addition, larger *F* and smaller *p* values also denote a more accurate regression model.

In this study, an IVM sustained-release bolus was prepared and optimized by RSM. The release kinetics of the IVM bolus was studied by in vitro dissolution tests (Chen et al., 2018; Wang et al., 2018), whereas the release patterns of the bolus were analyzed by model fitting of the in vitro release data (Feng, Li & Tan, 2017; Ramteke et al., 2014).

## Materials and Methods

### Materials

Ivermectin was purchased from Shandong Qifa Pharmaceutical Co., Ltd (Jinan, China). Soluble silicate glass (SiO_2_:Na_2_O, 3.4:1 m/m) was prepared in the Laboratory of Veterinary Pharmacology and Toxicology, Nanjing Agricultural University (Nanjing, China). Microcrystalline cellulose (MCC), starch, and low-substituted hydroxypropyl cellulose (LS-HPC) were kindly provided by Anhui Sunhere Pharmaceutical Excipients Co., Ltd (Hefei, China). We employed a mechanical pill press (AMHL-60) produced by Changzhou Aomuhalei Machinery Co., Ltd (Jintan, China) and a dissolution apparatus (SY-6D) obtained from Huanghai Drug Test Instruments Co., Ltd (Shanghai, China).

### Methods

#### Optimization of bolus formulation

The MCC, starch, and LS-HPC levels were the factors varied in the RSM analysis. The MCC levels considered were 8% and 10%, together with starch levels of 0.3% and 0.7%, and LS-HPC levels of 0.25% and 0.5%. The effects of these factors were tested by Box–Behnken design using Design Expert 8.0 (Stat-Ease, Inc., Minneapolis, MN, USA), as shown in Table 1. We prepared a mixture containing barium sulfate and soluble silicate glass (2:1, w/w), which were granulated and passed through a 12-mesh sieve after adding an appropriate amount of water to wet. Then, the particles were dried at 60 °C for 30 min, and further passed through a 14-mesh sieve after size stabilization. The bolus was compressed by applying a pressure of 750 kg/cm^2^ using the mechanical pill press, and then dissolved in a cup containing 900 mL dissolution medium at 39.5 °C under stirring at 100 rpm. The dissolution medium was prepared as described by Menke (Menke & Steingass, 1988), except that sheep rumen fluid was not added. The bolus formulation was optimized according to the dissolution time. According the above optimized formulation, an appropriate amount of IVM was added to a 12.6 g blank bolus. Then, the IVM-containing bolus was prepared using the method described above. The length, width, and height of the prepared IVM bolus were 28.12 ± 0.14, 16.1 ± 0.13, and 13.03 ± 0.05 mm, respectively. The bolus weighed 11.4842 ± 0.1675 g, contained 458.26 ± 6.68 mg of IVM, and its density was 1.95 g/cm^3^.

**Table 1:** Disintegration time measured in Box–Behnken design runs.

Std	Run	MCC (%)	Starch (%)	LS-HPC (%)	Disintegration time (d)
14	1	9	0.5	0.38	15
2	2	10	0.3	0.38	3
10	3	9	0.7	0.25	50
1	4	8	0.3	0.38	7
11	5	9	0.3	0.5	5
6	6	10	0.5	0.25	35
4	7	10	0.7	0.38	14
5	8	8	0.5	0.25	65
8	9	10	0.5	0.5	16
3	10	8	0.7	0.38	6
12	11	9	0.7	0.5	7
7	12	8	0.5	0.5	10
13	13	9	0.5	0.38	12
15	14	9	0.5	0.38	13
9	15	9	0.3	0.25	35

#### In vitro release kinetics of IVM bolus

Six IVM boluses used as parallel samples (*n* = 6) were dissolved using the United States Pharmacopoeia apparatus II at 100 rpm, maintaining the temperature of the dissolution medium at 39.5 °C. The dissolution medium consisted of 900 mL of artificial rumen fluid containing 4.5 g of sodium dodecyl sulfate (Ding et al., 2015). The preparation method of the artificial rumen fluid was according to Menke & Steingass (1988) and was slightly modified. Briefly, A solution: 13.2 g of calcium chloride dihydrate, 10.0 g of manganese chloride tetrahydrate, 1.0 g of cobalt chloride hexahydrate, and 8.0 g of ferrous chloride hexahydrate were dissolved in 100 mL of distilled water. B solution: 35.0 g of sodium bicarbonate and 4.0 g of ammonium bicarbonate were dissolved in 1,000 mL of distilled water. C solution: 5.7 g of sodium dihydrogen phosphate, 6.2 g of potassium dihydrogen phosphate, and 0.6 g of magnesium sulfate heptahydrate were dissolved in 1,000 mL of distilled water. The reducing solution consisted of 95 mL of distilled water, four mL of one M sodium hydroxide, and 625 mg of sodium sulfide 9-hydrate. A total of 400 mL of distilled water, 0.1 mL of A solution, 200 mL of B solution, 200 mL of C solution, and 40 mL of reducing solution were sequentially added to form the artificial rumen fluid. The samples were collected every day until the end of the experiment, whereas the dissolution medium was changed every day. The samples were analyzed using a well-established method (Alvinerie et al., 1999) with slight modifications. Briefly, 0.5 mL of the deliquated dissolution medium was mixed with one mL of ethyl acetate for 3 min. Then, the solvent–sample mixture was centrifuged at 13,225 × *g* for 10 min. The supernatant was transferred to a tube and the extraction was repeated once. The combined supernatants were dried under a stream of nitrogen (Anpel Laboratory Technologies, Shanghai, China), and then re-suspended in 100 μL of a solution of *N*-methylimidazole (Aladdin, Los Angeles, CA, USA) in acetonitrile (1:1, *v/v*) (De Montigny, Shim & Pivnichny, 1990). Derivatization was initiated by addition of 150 μL of trifluoroacetic anhydride solution (Aladdin) in acetonitrile (1:2, *v/v*). After reacting for 30 min, the solution was centrifuged at 13,225 × *g* for 10 min at −4 °C. Finally, an aliquot (20 μL) of this solution was injected directly into a High performance liquid chromatography (HPLC) system (Waters, Milford, MA, USA). HPLC analyses were carried out using a reverse-phase Eclipse XDB-C18 column (Φ = 5 μm, 4.6 × 250 mm) and a water/methanol (3:97, *v*/*v*) mobile phase at a flow rate of 1.0 mL/min, at a temperature of 40 °C. IVM release was monitored with a spectrofluorometric detector (Waters 2475; Waters, Milford, MA, USA) at excitation and emission wavelengths of 365 and 475 nm, respectively. The cumulative amount and percentage of released IVM were determined according to the corresponding in vitro release data.

#### Statistical analysis

The release pattern of the IVM bolus was analyzed using several standard kinetic models including zero order, first order, Korsmeyer–Peppas, Higuchi, Hixson–Crowell, and Weibull models (Feng, Li & Tan, 2017; Ramteke et al., 2014). The in vitro release data obtained from the dissolution medium were fitted to the above kinetic models using the respective formulas, and the corresponding dissolution kinetic curves were constructed. The best fitting model was identified according to two statistical parameters, the regression coefficient (*R*^2^) and the Akaike Information Criterion (AIC). In particular, after comparing the *R*^2^ and AIC values obtained for the different kinetic models, it was found that the best model of the dissolution kinetics exhibited *R*^2^ values near to 1 and the lowest AIC value. Statistical analyses were performed using Microsoft Office Excel® (Microsoft Corporation, Redmond, WA, USA) and Origin 9.1 (OriginLab, Hampton, MA, USA).

## Results

### Formulation optimization

**Figure 1:**
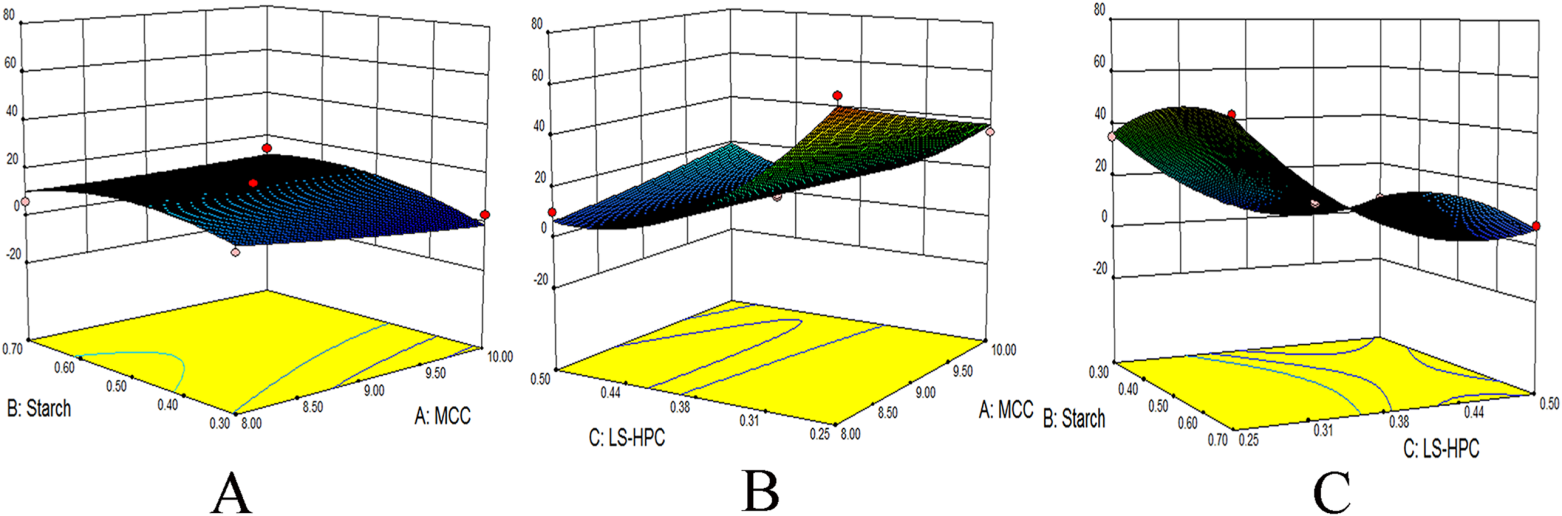
3D contour surfaces of bolus disintegration time. Effect of MCC-starch (A), MCC-LS-HPC (B), and starch-LS-HPC (C) interactions on disintegration time.

Different MCC, starch, and LS-HPC contents resulted in different dissolution times of the corresponding boluses (Table 1). The longest and shortest dissolution times were 65 and 3 days, respectively. ANOVA was used to assess the adequacy of a quadratic model selected to represent the experimental data (Table 2). LS-HPC and starch were found to have extremely significant (*p* < 0.01) and significant (*p* < 0.05) effects on the bolus dissolution time, respectively. The interactions between two of the three independent variables (MCC, starch, and LS-HPC levels) were analyzed using 3D contour plots (Fig. 1). The optimized bolus formulation contained 8% MCC, 0.5% starch, and 0.25% LS-HPC.

**Table 2:** ANOVA for response surface quadratic model of disintegration time data.

Source	Sum of squares	d*f*	Mean square	*F* value	*p*-value prob > *F*	
Model	4,600.82	9	511.2	23.47	0.0014	Significant
A-MCC	50	1	50	2.3	0.1902	
B-Starch	91.12	1	91.12	4.18	0.0962	
C-LS-HPC	2,701.12	1	2,701.12	124	0.0001	
AB	36	1	36	1.65	0.2549	
AC	324	1	324	14.87	0.0119	
BC	42.25	1	42.25	1.94	0.2225	
A^2	1.85	1	1.85	0.085	0.7823	
B^2	158.01	1	158.01	7.25	0.0431	
C^2	1,125.39	1	1,125.39	51.66	0.0008	
Residual	108.92	5	21.78			
Lack of fit	104.25	3	34.75	14.89	0.0636	Not significant
Pure error	4.67	2	2.33			
Cor total	4,709.73	14				

### In Vitro release kinetics of IVM Bolus

**Figure 2:**
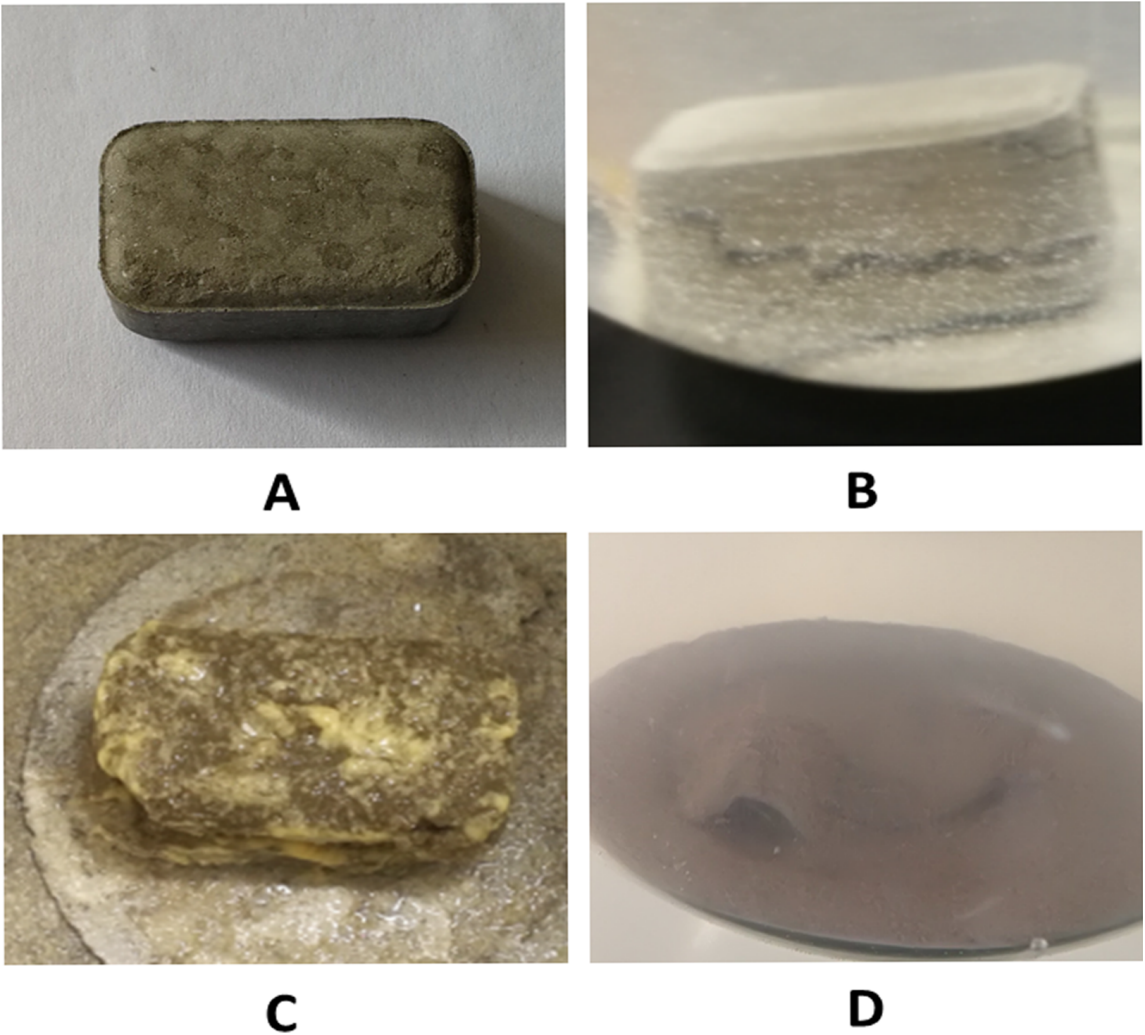
The appearance changes of IVM bolus during in vitro release tests. (A) IVM bolus formulation. (B) A small crack appeared after IVM bolus release in the dissolution medium at 4 h. (C) IVM bolus was placed in the dissolution medium for 30 days. A minor portion (edges and corners) of the IVM bolus fell off and dissolved, but its shape was basically intact. (D) A small part of the shell of IVM bolus was left in the dissolution medium at 60 days (Photo credit: Xiang Chun Ruan).

The 15 tests with different compositions were optimized by Box–Behnken design for the formulation of IVM bolus. The optimized bolus formulation contained 8% MCC, 0.5% starch, and 0.25% LS-HPC. IVM boluses optimized with RSM were studied by in vitro release kinetics. Six IVM boluses (six replicates) were dissolved in the dissolution medium. Almost all IVM boluses showed cracks. Only the size of the crack might be a little different. Shown in Fig. 2B was one of the six cracking boluses after 4 h immersion in the dissolution medium. The appearance of cracks in the dissolution medium of IVM bolus was primarily the result of the action of disintegrating agents. However, the bolus maintained a compact shape, and its content was released through the cracks. The bolus exhibited sustained IVM release for more than 60 days (Fig. 2).

**Figure 3:**
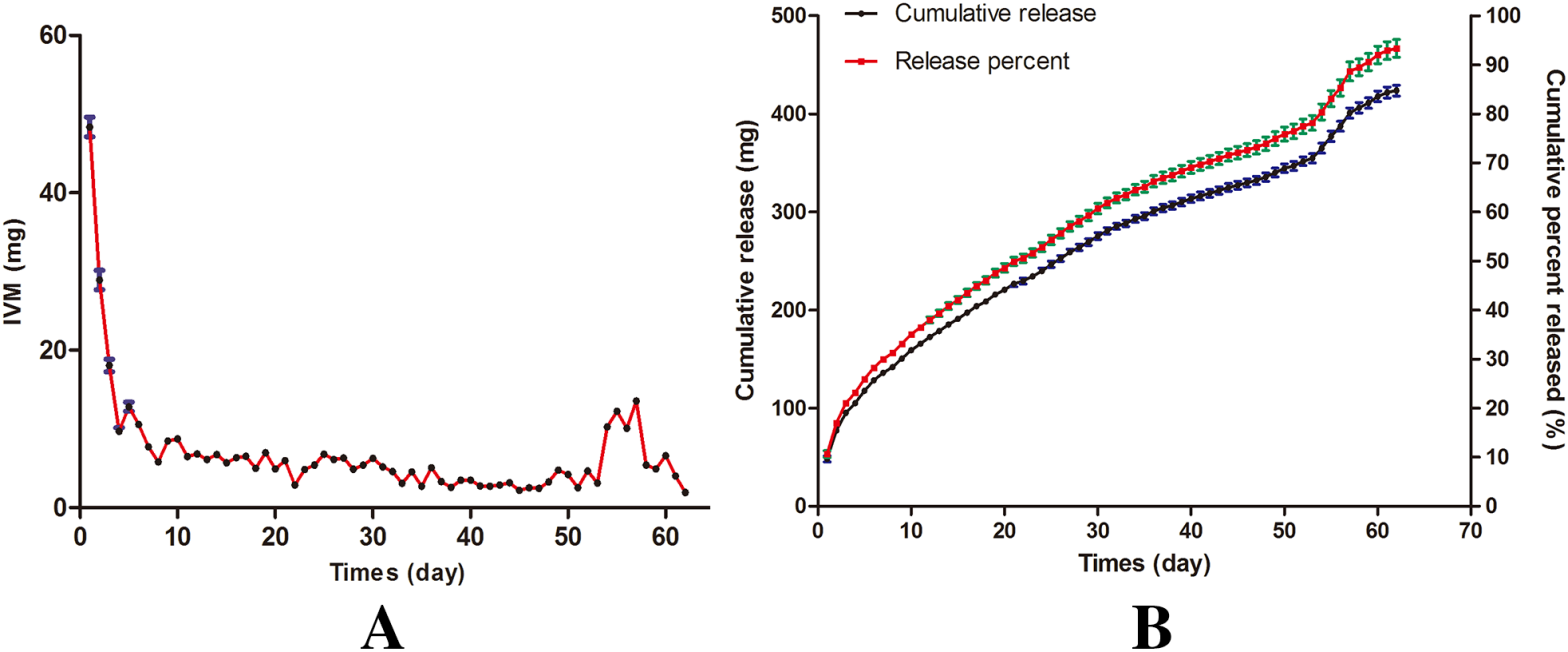
In vitro release kinetics of IVM bolus. (A) IVM released in vitro. (B) Cumulative and percent release of IVM.

A significant burst effect was observed, with 48.37 ± 3.04 mg IVM released in the first day, followed by a slower release. A sustained-release of two to eight mg IVM/day occurred from day 7 to day 53. The released amount of IVM increased to ten to twelve mg/day from day 54 to day 57, and then declined until the release was completed (Fig. 3A). The total released amount of IVM was 423.72 ± 5.48 mg, corresponding to 92.52 ± 1.20% of the initial content (Fig. 3B).

### Statistical analysis

**Figure 4:**
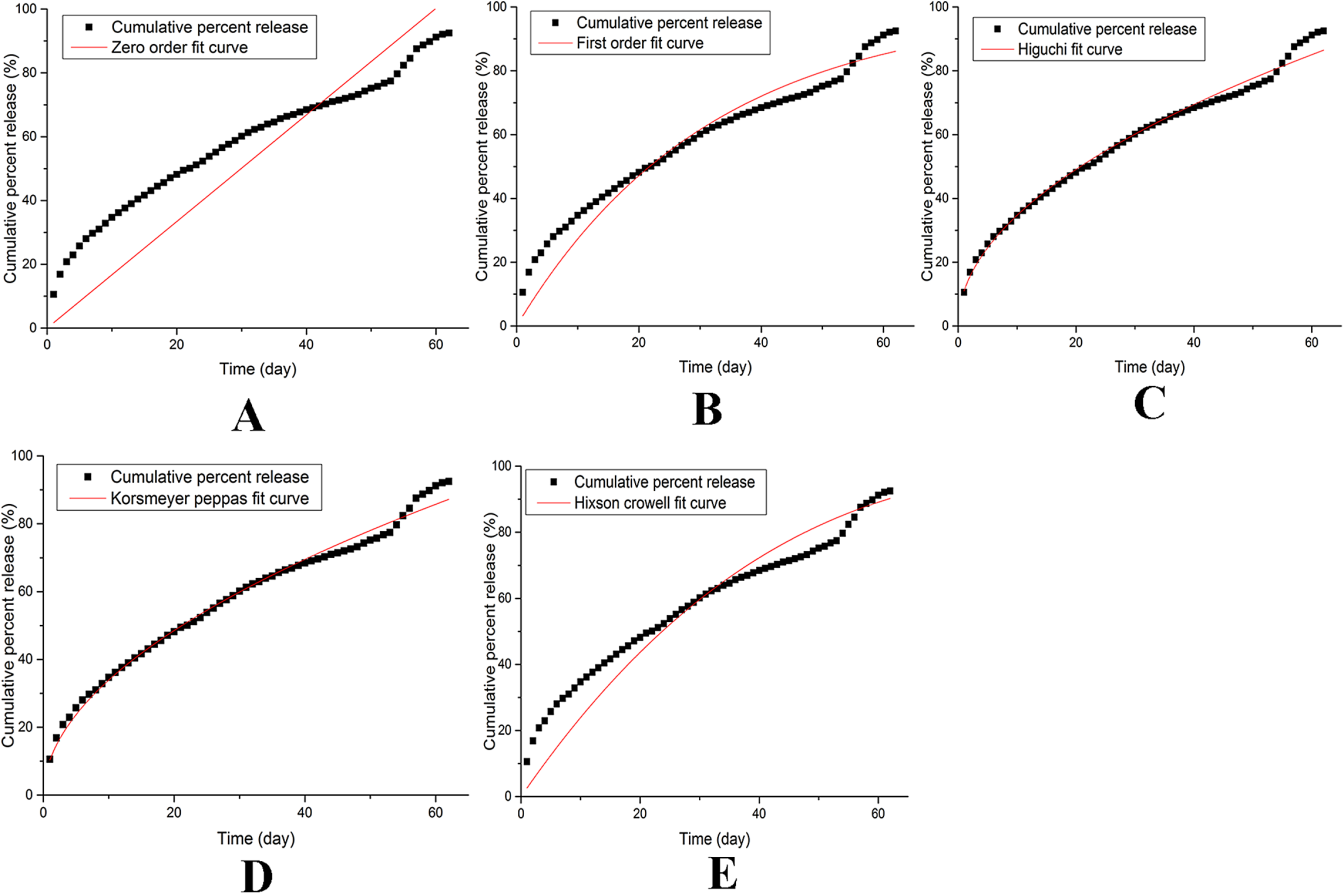
Models of release kinetics of IVM bolus in dissolution medium. (A) Zero-order. (B) First-order. (C) Higuchi. (D) Korsmeyer–Peppas. (E) Hixson–Crowell.

Different dissolution kinetic models were applied to fit the dissolution data (Fig. 4), the calculated *R*^2^ and AIC parameters corresponding to each model are shown in Table 3. We found that the Weibell model failed to fit the data, whereas a satisfactory goodness of fit was obtained for the Korsmeyer–Peppas model. In particular, this model provided the best fit to the dissolution kinetic data, as it produced an *R*^2^ value close to 1 and the lowest AIC among the tested models (Table 3). Moreover, the *n* parameter (0.5180) of the Korsmeyer–Peppas model was between 0.45 and 0.89. These results thus demonstrate that the release of IVM occurs through a diffusive erosion mechanism (Korsmeyer et al., 2012).

**Table 3:** Dissolution kinetic model fitting parameters.

Model	Equation	Parameters	*R*	AIC
Zero order	*y* = *k* × *x*	1.6709	0.6653	563.54
First order	*y* = 100 × [1 − exp(−*k* × *x*)]	0.0319	0.9360	460.94
Higuchi	*y* = *k* × *x*^0.5^	10.9806	0.9885	356.53
Korsmeyer–Peppas	*y* = *k* × *x*^*n*^	10.2859, 0.5180	0.9891	352.47
Hixson–Crowell	*y* = 100[1 − (1−*k* × *x*)^3^]	0.0087	0.8969	490.52
Weibell	*y* = 100 × {1 − exp[−(*x*)^*b*/*a*^]}	Failed	Failed	Failed

## Discussion

Silicate soluble glass (one component of the bolus) is a biomaterial with good biocompatibility, is not harmful to animals over the long term (Njanja, Bell & Westcott, 1998). Glass particles within the bolus formulation disintegrate, but some of the particles are resorbed in the body right up to complete degradation, and some of particles are not resorbed in time and can be excreted with feces (Hench, 2006). Silicate glass within a bolus given by oral administration appears to be a safe method.

The size, shape, and density of ruminant bolus have been investigated by Telfer and Cardinal (Cardinal, 1985; Telfer, 1984). The size and the shape of the bolus changes according to the ruminant animal. However, the bolus density is the most important factor determining its transit time in rumen. Owing to its high density, barium sulfate is used as barium meal for X-ray detection. It has been reported that the sustained-release bolus can contain up to 70–80% barium sulfate (Wood, Toothill & Dietz, 1994). Therefore, barium sulfate was used in this work to regulate the density of the IVM bolus. The density of deworming tablets was reported to be approximately 3.0 g/cm^3^ (Marsten, 1962), whereas the density of formulations developed for captive ruminants was lower, 1.8 g/cm^3^ (Vandamme & Ellis, 2004). Therefore, the density of the IVM bolus (1.95 g/cm^3^) appears suitable to remain in rumen for an appropriate period of time.

Ivermectin bolus cracked in the dissolution medium and released after 10 days. The dissolved components of the IVM bolus remained relatively constant. It maintained a relatively stable release. The flatter portion of the curve is shown between 10 and 53 days. Although in vivo pharmacokinetic studies have not been conducted, the release levels of IVM bolus from 10 to 60+ days are likely to be biologically active. Therefore, we assume that IVM bolus could maintain to be in bioactive amounts over 60 days. After a relatively stable release of the IVM bolus, the dissolution of IVM is accelerated and the amount of IVM release increases due to the final disintegration of the bolus. The peak in the release curve was at 50–60 days. The small external shell remained at 60 days. IVM release was decreased until it was not being detected after 62 days.

The cracks in the IVM bolus were the result of the action of the disintegrants (starch and LS-HPC). The IVM was released through the crack and mainly released in a diffusion style at an initial stage. Afterwards, it might be dominated in an erosion mode and achieve a relatively stable release. The presence of gaps determines the release kinetics of IVM bolus. Different disintegrants, such as sodium carboxymethyl cellulose and sodium carboxymethyl starch, have different effects on the release kinetics of IVM bolus.

The present bolus exhibited sustained IVM release for more than 60 days in vitro, with a cumulative percentage of released IVM higher than 90%. About 10% of the IVM content (48.37 ± 3.04 mg) was released in the first day, which is ten times higher the IVM therapeutic dosage for a sheep of 25 kg body weight. Extended parasite exposure of IVM may improve its efficacy against anthelmintic-resistant parasites (Alvarez et al., 2015). Parasitological data tend to demonstrate that the oral route is more effective against intestinal nematodes (Mckellar & Benchaoui, 1996). Significantly higher IVM concentrations in abomasal content have been measured after intraruminal treatment compared with subcutaneous injection in sheep (Lloberas et al., 2012). However, IVM injection via the subcutaneous route has been shown to elicit transient pain in goats (Njanja, Bell & Westcott, 1985), so oral administration of IVM would be more appropriate. In cattle or goat industries, long-acting products are used widely because they: (i) can treat existing infections by parasitic nematodes and prevent new infections; (ii) need substantially fewer resources to treat animals due to the reduced frequency of administration, livestock handling, and restraint (labor and equipment) (Forbes, 2013). Hence, the novel bolus is possible to become a drug-delivery device for anthelmintic agents in cattle and goat industries.

## Conclusions

The optimized bolus formulation contained 8% MCC, 0.5% starch, and 0.25% LS-HPC, as determined by RSM. The optimized IVM bolus exhibited sustained-release for more than 60 days. The in vitro cumulative release and released percentage of IVM were 423.72 ± 5.48 mg and 92.52 ± 1.20%, respectively. The Korsmeyer–Peppas model provided the best fit to the dissolution kinetic data. The release of IVM followed a diffusive erosion mechanism. Further research is needed to investigate the correlation between the in vitro/in vivo release and the clinical treatment effects of IVM boluses.

## Supplemental Information

10.7717/peerj.5418/supp-1 Supplemental Information 1 Disintegration time measured in Box-Behnken design runs.

10.7717/peerj.5418/supp-2 Supplemental Information 2 In vitro release of IVM bolus.

10.7717/peerj.5418/supp-3 Supplemental Information 3 In vitro cumulative release of IVM bolus.

10.7717/peerj.5418/supp-4 Supplemental Information 4 In vitro cumulative percent release of IVM bolus.

## Additional Information and Declarations

### Data Availability

The following information was supplied regarding data availability: The raw data are provided as Supplemental Files.
